# Lectures on the Diseases of the Urinary Organs

**Published:** 1842-07

**Authors:** 


### Art. XIV.

*Lectures on the Diseases of the Urinary Organs.* By Sir Benjamin C. Brodie, Bart. f.r.s., Serjeant-surgeon to the Queen. Third Edition, with Alterations and Additions.—*London,* 1842. 8vo, pp. 379.

Under ordinary circumstances, when a book has reached a third edition, public opinion has stamped its value, and the praise or blame of the critic is likely to pass unheeded; but the work before us is, to a certain extent, rewritten, and the author has been able to bring to bear a large mass of experience obtained subsequently to the publication of the previous edition upon certain points still undecided, but of great importance; and these form a legitimate subject for examination in the present notice.

In reviewing the productions of distinguished men, it becomes us to differ with humility, to controvert with diffidence, but to offer our own opinions with frankness and honesty. Sir B. Brodie is the first surgeon in a country which has produced some of the greatest names of modern surgery; in his favour popular opinion and the judgment of professional rivals have concurred; and without any of that warmth of manner which excites enthusiasm and calls forth troops of friends, he is placed by common consent at the head of his profession. It has been the lot of many men who have occupied that position to have been forgotten when the grave closed over them; they had contributed nothing to posterity, and by posterity they were not remembered. The sagacious practitioner is not always the successful author, and often leaves nothing by which those who follow him can judge whether success depended on good fortune rather than merit, on personal rather than professional recommendations. It is not thus with Sir B. Brodie; and when as the successful practitioner he is no longer remembered, he will be known as the author of two works, which, on the subjects to which they relate, are the best we yet possess.

Many circumstances have concurred in our day to extend more widely than formerly a knowledge of the literature of our profession; and the standard works of all countries are now studied by the well-educated surgeon. There may thus be found in contemporary publications an acquaintance with the experience of other times and other countries more complete and extensive than that which is exhibited in the work at the head of our present article; but that work possesses merits of a higher kind than any literary excellence can give, inasmuch as the writer thoroughly understands the diseases of which he has treated, and is acquainted with the best methods of cure; and instead of reading the works of other men in order to write a book, he has from his own experience furnished materials for extending that particular department of medical science to the cultivation of which his life has been devoted.

The earlier portion of the work before us is devoted to the consideration of stricture of the urethra. As compared with the last edition, we do not see in this any important alterations to call for remark; and as our last number contains a review of several works on that affection, we shall offer only a very few observations upon it here. We entirely agree with the author that in a great majority of cases any means with which we are acquainted are insufficient for the *cure* of ordinary organic stricture: by which we mean a contraction, dependent upon the development of indurated matter, in the submucous tissue. Yet how many men there are in this metropolis at the present moment who are quite ready to undertake to *cure* any case of stricture which may come in their way! Where the disease is recent, where the new matter has not acquired great density, there can be no question that the pressure of a bougie may be sufficient to cause its removal, just as an induration of an inguinal gland may be dissipated by the pressure of a truss; but then great care in the application of the pressure is necessary, or the evil may be increased. The rule for the guidance of the practitioner should be this: Let the bougie be introduced as often as it can be borne without occasioning much and increasing pain or inconvenience; and the same circumstances must determine the length of time during which it should be retained in the canal. There is an objection, however, to too rapid or "vital" or "permanent" dilatation, as it has been sometimes termed, and it is—that the canal more quickly resumes its contracted condition than when it is more gradually effected.

With reference to the treatment by *nitrate of silver*, we do not think our author has devoted sufficient space to the subject, taking into the account the potency of the remedy, and considering its importance. Whatever he may think of this mode of treating stricture, it is necessary, at least we think it would have been better, that the *pros* and *cons* should have been more fully stated, because the enthusiasm once excited in its favour by Home is not quite extinguished. It is hardly correct to say that cauterization was first proposed by Mr. Hunter. It is certain that caustics were used by Alphonso Ferri, by Paré, by Soyseau on Henry IV. of France, and by our own Wiseman, long before the time of Hunter.

Sir B. Brodie almost limits the use of the nitrate of silver to the treatment of those strictures which are attended with much irritability or spasm; and certainly the effect is then, in many cases, very remarkable. Sometimes a single application is sufficient to dissipate the irritability even when it has been so great as to occasion spasm, whenever the urine came in contact with it, as well as to remove any induration which may exist: just as the hardened edges of an irritable ulcer will give way to the application of lunar caustic over the surface.

So much of inaccuracy pervades the ideas commonly entertained with regard to cauterization of the urethra, that we are induced to make a few remarks, with the hope of correcting the evil. One of two objects is to be attained by the application of nitrate of silver upon the mucous membrane of the urethra: either a modification of an exaggerated sensibility, or a destruction of substance. With respect to the attainment of the first object, it is no longer a matter of doubt. With regard to the second, some observations are necessary. The induration is rarely, if ever, confined to the mucous membrane, which in many cases appears to have undergone little or no change; it is found to be very much confined to the submucous tissue. Now if the nitrate be employed for the purpose of effecting the destruction of the indurated mass, it must be evident that before this can be accomplished, there must have been complete destruction of the entire thickness of the mucous coat, there must have been destruction of the submucous hardened tissue, and there must be a subsequent contraction of the surface in the work of cicatrization: in fact there will be a *new* contraction which may require for the remainder of life the daily use of a bougie to overcome. We apprehend, therefore, that the plan of treating stricture by destruction, through the agency of nitrate of silver, is one which must be abandoned. Although this is the principle upon which that plan of treatment is supposed to be founded, we doubt whether it has often been carried out. We think that the mode in which it has been attempted to carry it out can only very rarely have accomplished the desired object, and for these reasons: the mucous membrane of the urethra is as thick as that which lines the cheeks; now if a piece of lunar caustic be kept in contact with the latter membrane for a minute, a slough will be formed; in a few hours it will be thrown off, and if in a couple of days afterwards the part be examined, no change of texture will be detected, and many applications upon the same point at short intervals will be required to produce any considerable change of structure. Although, then, we do not deny that the complete destruction of the mucous membrane may be accomplished by the repeated application of lunar caustic, we believe it to be happily a rare occurrence.

As to *cutting instruments*, we have always regarded their employment with much apprehension: the chance of injury to healthy parts is so great; the probability of the incision, when made, being confined to the proper point, is so small; the means of attaining the object by safer agents are so obvious, that we wonder the lancetted stilette is any longer found in the armamentarium of prudent surgeons. If, says our author, any cases occur in which this method may be useful, they are undoubtedly very few in number.

We would desire to impress upon young surgeons the following advice by one so competent to advise on the subject:

"But I cannot too strongly impress it on your minds, that in the treatment of stricture you ought not to use violence under any circumstances. Your success in the cure of this disease will depend very much on your attending to this important rule. Whether you use a bougie, or a sound, or a catheter, let the instrument be held lightly, and as it were loosely, in your hand; it will then, in some measure, find its own way in that direction in which there is the least resistance; whereas if you grasp it with force, the point can pass only where you direct it, and it is just as likely to take a wrong course as a right one. A stricture will invariably resent rough usage: it will yield to patience and gentle treatment." (p. 68.)

In Chap. vi. there is a section of great value, showing that diseases of the kidney may be manifested prominently by symptoms affecting the bladder, while the ordinary symptoms of diseased kidney are marked: "Whoever is much engaged in this branch of surgical practice will meet with a number of facts which cannot so well be explained on any other hypothesis, and which collectively form such a mass of circumstantial evidence as is almost irresistible in favour of the opinion that the worst symptoms of irritable bladder may occur as a consequence of disease of the kidney, the bladder itself and the organs in immediate connexion with it having been free from disease in the first instance." (p. 125.) Cases are mentioned which seem to afford sufficient evidence as to the existence of symptoms referred to the bladder and urethra in some cases of diseased kidney: "But with my present experience I am led to this further conclusion, that a very large proportion of the cases which have usually been confounded together under the general appellation of irritable bladder are really of this description." (p. 131.) But then it is equally certain that the kidney has been found much diseased while the bladder was free from trouble. "The question therefore arises, in what particular cases of renal disease it is that the secondary affections of the bladder are likely to occur," and to this point all our inquiries should tend:

"I have already explained that where the urine is overloaded with acid, showing itself in the form of lithate of ammonia, or brown or red sand, or where, being alkaline, it deposits crystals of the triple phosphate of ammonia and magnesia, it acts as a stimulus to the parts with which it comes in contact, and that an irritable state of the bladder is the consequence. But there is no reason to doubt that other unhealthy secretions of urine may produce the same result; and I am much inclined to believe that such is the real explanation of the affection of the bladder in the cases which are now under our consideration. In such of them as have fallen under my observation, the urine has been always altered from its healthy condition, and its sensible qualities may be described as follows: There is usually a copious secretion, the specific gravity being below the ordinary standard. But there is some variety in this respect; and I have known the specific gravity to be as high as 1030. When tested with litmus paper, it is generally found to be slightly acid, but occasionally it is alkaline, or it is sometimes alkaline and sometimes acid, and, as I shall explain hereafter, the disposition in it to become alkaline increases as the disease advances. When first voided, the secretion is of a pale yellow colour, opaque, and turbid, sometimes having minute flakes of lymph floating in it. On the addition of nitric acid, or on exposure to heat, there is an abundant coagulation of albumen. When allowed to remain at rest, there is a deposit of opaque matter, and not unfrequently of pus. The urine is always albuminous, but quite different in appearance from that which is secreted in the cases which were first described by Dr. Bright, and to which the attention of physicians has been of late years so much directed. The albuminous matter seems to be mechanically suspended, and not intimately blended and assimilated with it; as if the kidney were in a state of chronic inflammation, secreting urine from one set of vessels, and serum or even pus from others. Such, probably, is the real nature of the disease when once established, whatever it may have been in its origin." (pp. 131-3.)

Though we must admit the practical force of much of our author's reasoning on this very important subject, it is not to our minds conclusive; and we think further opportunities of observation will serve still more to widen the question. We apprehend that many different affections of the kidney may produce a similar condition of the functions of the bladder. We have seen symptoms of disease of the bladder in what appeared to be simple nephritis, the urine having undergone little or no change; in calculus of the kidney, where there was for years no pain in the lumbar region: they may also happen in pyelitis, as well as in chronic albuminous nephritis. As the disturbance in the bladder may occur under such varied circumstances, and may also be absent under each of them, it must be evident to everybody that, though Sir B. Brodie has done much to throw light on this obscure point of pathology, still more remains to be done to determine what particular abnormal condition of the kidney manifests itself by the development of those remarkable symptoms in the bladder.

Besides the case cited from Morgagni there is another (Ep. lxii. sect. 5) observation by Harder, related by Bonnet, (Sepulchretum, lib. iii. sect. xxvii. obs. 10.) A paraplegic child, three years old, was attacked with convulsions, and showed by its gestures that it suffered violent pain in making water, without ever having complained of renal pains. After death the bladder was found to be healthy, but a large quantity of sand could be squeezed out of the mammillæ. Lowdell (Mem. Med. Soc. vol. i. p. 319) describes the case of a woman of twenty-two, who suffered much from pain in the bladder, but who never complained of pain in the region of the kidneys. The calices of both kidneys contained large irregular calculi. Mr. Howship mentions a case (Urinary Organs, p. 40) where there was great pain at the neck of the bladder, with frequent desire to make water, the urine containing thick mucus. The bladder was found perfectly healthy; the right kidney was converted into a purulent sac. It is no doubt rare to find similar facts; but it is not so rare to find considerable disease of the kidneys in persons who never complained of lumbar pains, and who had suffered from irritation in the bladder. How many persons have undertaken to extract calculi from the bladder after carefully examining into the evidence of renal disease, and satisfying themselves that they had nothing to fear in that quarter—have well performed the operation, and quickly lost their patient, and after death have discovered inflammation, pus, or calculi in the kidney! In the present state of our knowledge the difficulty is to point out what particular condition of the kidneys it is which determines the prominent bladder-symptoms. It is clear that it must be some element common to the several affections which have been alluded to, and probably it is a particular form of inflammation.

There are not wanting books to prove that *chronic enlargement of the prostate* is a *very curable* disease; and as patients are apt to take their opinions in such affairs from those who promise most, and as young practitioners are not unfrequently led to encourage hopes which end in disappointment, we would particularly direct their attention to the following remarks, in every word of which we concur.

"At different periods of human life different changes take place in the condition of the organs of which the system is composed; and none of these are more remarkable than those which show that the individual has entered on that downward course which is to end in dissolution........... When the hair becomes gray and scanty, when specks of earthy matter begin to be deposited in the tunics of the arteries, and when a white zone is formed at the margin of the cornea, at the same period the prostate gland usually—I might, perhaps, say invariably—becomes increased in size. This change in the condition of the prostate takes place slowly and at first imperceptibly, and the term, chronic enlargement, is not improperly employed to distinguish it from the inflammatory attacks to which the prostate is liable in earlier life." (p. 152.)

This is a condition almost as little under the dominion of art as the deposits in arteries.

"If you bear in mind that the chronic enlargement of the prostate gland to which I called your attention, is not an accidental disease, but one of a series of natural changes which the system undergoes after the middle period of life, you will not be surprised to find that it is but little under the dominion of art. .............. We are not acquainted with any method of treatment which is capable of restoring the gland to its original condition............. Nevertheless, in these cases, much may be done by means of proper surgical treatment. The prostate of a man advanced in life cannot be rendered like that of a young man, any more than his gray hairs can be converted into black; but the train of evils which the enlarged prostate produces by its influence on the urinary organs may be in some instances altogether prevented, and in others very much diminished, so as to remove the patient from a state of extreme and even immediate danger to one of comparative security." (pp. 173-4.)

There is a caution with respect to *retention of urine from enlarged prostate*, to which we particularly direct attention.

"There are some [cases] in which much discretion is required in resorting to the use of the catheter. What I am about to state is not an opinion formed hastily, but a deliberate conclusion to which I have been led, after having had for many years no small share of experience in the treatment of these disorders, as well as considerable opportunities of investigating the morbid appearances which they leave behind them in the dead body. If in a case of chronic enlargement of the prostate the patient has been allowed to go on for two or three years, or longer, without the use of the catheter, and in consequence of this neglect the quantity of residuary urine in the bladder has gradually increased, so that at last one or two or more pints are accumulated in it, the kidneys having at the same time become diseased, the introduction of the catheter according to the rules formerly laid down, so as to empty the bladder two or three times daily, is likely to be injurious rather than beneficial. The patient is, it is true, relieved of many of his distressing symptoms. He is no longer tormented by a frequent desire to void a small quantity of urine, nor by an involuntary dribbling of urine during the night; nor does he suffer the uneasy sensations which, in a greater or less degree, always attend an over-distended bladder; but in the course of a few days it is observed that he avoids his usual exertion, that he seems languid, and loses his disposition to take food. Then the other symptoms of disease of the kidney, which were imperfectly developed before, become distinctly marked, and he gradually sinks and dies at the end of a month or six weeks from the time of the catheter being first employed." (pp. 189-90.)

Our own experience enables us to confirm the correctness of this statement; but why it happens is not easy to say, though it is probably owing to the sudden removal of pressure from distension of the kidney, to its inability at once to adapt itself to its altered circumstances, and to a consequent more or less complete suppression.

"Here then arises an important practical question. The patient has no chance of recovery without the use of the catheter. Are we to leave him to his fate? or are we to empty his bladder at certain intervals, at the risk of hastening the period of his dissolution? I have no doubt that we may, in many instances at least, obtain the good and avoid the evil by a slight modification of the treatment. Let the catheter be introduced at first so as to draw off only a portion of the contents of the bladder, and let several days be permitted to elapse before it is completely emptied; care being taken at the same time to uphold the general health by the exhibition of ammonia, quinine, and other tonics, exhibited according to circumstances, and combined with the prudent use of wine or brandy, and a plain but nutritious diet." (pp. 191-2.)

The portion of the work devoted to stone in the bladder is particularly interesting. Our author adheres to the commonly received, but to our mind incorrect, opinion that among the lower classes children are *much more* liable to calculi than grown up people. "You know how large a proportion of hospital patients admitted are children." Now how stands the fact? We take one collection of cases amounting to 5967, and we find them thus divided: under 14, 3031; above 14, 2396 cases; no great difference. But when we dissect the returns made up from different countries, we are struck with the great variation in the proportion of children to adults in the different returns.

| | | | | | |
|---|---|---|---|---|---|
| Thus of 356 operations | reported by | Dupuytren | 97 only occurred | in children | under 15. |
| 1629 " | " | Saucerotte | 1195 | " | " |
| 478 at Norwich | " | Marcet | 227 | " | " |
| 666 at Naples | " | Renzi | 315 | " | " |
| 643 " | " | " | 321 | " | " |
| 469 St. Petersburg | | | 357 | " | " |
| 175 Austria | | | 55 | " | " |
| 239 Bavaria | | | 116 | " | " |
| 106 Bohemia | | | 28 | " | " |
| 145 Denmark | | | 14 | " | " |
| 42 Egypt | | | 1 | " | " |
| 2822 France | | | 1347 | " | " |
| 1058 Lombardy | | | 796 | " | " |
| 49 Rome | | | 10 | " | " |
| 140 Sardinia | | | 97 | " | " |
| 127 Ulm | | | 64 | " | " |

Taking large numbers it would appear that calculus is little if at all more frequently found among the children than the adults who are treated in hospitals. If we examine into the frequency of its occurrence in children's institutions, we are struck with the comparative immunity from it enjoyed by them. In 27 years 1151 children were admitted into the Foundling hospital, and in the same time only three cases of stone occurred there. In the Military Asylum at Chelsea, out of 6000 admissions not one case was furnished. In the Hôpital des Enfans, at Paris, where 3000 children are received annually, during the last 7 years the average has been under four. At the St. Marylebone workhouse, where there has been on an average during many years between 400 and 500 children, there has not been a single case of stone either among them or the aged persons during the last twenty years.

There is something very obscure about the formation of calculi: why they prevail so extensively in some districts, so unfrequently in others; why London should be so free from them, while in Norfolk they are so rife; why in particular localities the majority of sufferers are children, while in others they are a small minority,—are matters which in the present state of our knowledge are inexplicable. The numerical results show that infancy is the period of life in which stone in the bladder is most frequently observed, though the comparative frequency has been exaggerated. Mr. Cross attributes the frequency of the occurrence of stone in the bladder in Norfolk and Suffolk to the influence of the cold north-east winds, which prevail in those counties, over the functions of the skin. This opinion may be reconciled to that of Magendie, who endeavoured to account for the frequency of calculous disorders in old men by the diminished temperature of the body, which contributes to favour the concretion of uric acid.

"There is a class of cases which being of rare occurrence do not seem, in the present state of our knowledge, of much practical importance, but which I am unwilling to leave altogether unnoticed, especially as they exhibit a phenomenon of much interest in pathology." This is the disintegration or spontaneous breaking down of stone in the bladder. A case is related from Heister, where it was supposed to have been brought about by the use of mineral water—another from Dr. Prout where no mineral water had been used—and two from Mr. Cross, in one of which it is inferred that the breaking down had been the result of a jolting on horseback, and two or three others. Sir B. Brodie says, "I give you these facts without any comment. Future observations are required to enable us to give a satisfactory explanation of them." Facts of this kind are not however of such rare occurrence, neither are they of such recent observation as seems to be thought. They have been observed by Borrich; Detharding, (Haller's Theses, 110;) Geoffroy, (Acad. 1739;) Whytt, (Edin. Essays, vol. vi. p. 263;) Velpeau, (lib. iv., cap. 37, p. 333;) Deschamps Morand, (Mém. de l'Acad. 1740-1;) and Rousseau and Civiale.

In some cases it occurred during the use of alkalis or acids; in others without any plan of treatment; in others "by rubbing against each other;" in others by contraction of the bladder upon them. Each of the alleged causes applies only to a small number of cases, while in similar or identical cases the agent has been without effect. Thus much we know—that a disaggregating influence was exercised on calculi submitted to the action of Vichy water—that a bladder containing calculi is often hypertrophied—that it acquires an increase of contractile power—and that a certain pressure may thus be exercised on the contained calculi. Covillard and Fabricius Hildanus mention the cases of some patients who could hear the calculi rubbing against each other in the bladder. In a case described in the Phil. Trans. 1731, the patient's bladder contracted and produced a sensation of something breaking in it, and the next minute he passed many fragments with the urine.

Before the operation for extracting the stone be undertaken, it is proper that every probable means of ensuring success should be taken. And it is therefore very important to establish what are the circumstances, if any, which should induce the surgeon to refrain from the attempt to remove a calculus from the bladder. None of those circumstances demand more serious attention than the state of the kidney.

"Success in lithotomy must undoubtedly depend in a great degree on the manual dexterity of the surgeon, and on the mode in which the operation is performed; but it depends still more on the condition of the patient with respect to his general health, especially on the existence or non-existence of organic disease............. Before determining on lithotomy, you have no more important duty to perform than that of enquiring into the state of the kidneys. ........... One thing to be especially attended to with a view to a correct diagnosis, is the state of the urine. The urine may be alkaline, and thus in an unnatural state, and yet the kidneys may be free from organic disease and the patient a proper subject for the operation. It is purulent and turbid urine loaded with albumen by which your apprehensions as to the result of an operation will be chiefly excited." (pp.243-4.)

Mr. Edwin Lee* does not seem to regard the existence of such organic disease as a bar to lithotrity. "In some cases of this description," he says, "lithotrity might be had recourse to with advantage, the shock to the constitution being comparatively small, and the irritation from this operation being less likely to extend to the diseased organ." We are, however, of Sir B. Brodie's opinion "that a small shock to the system will sometimes destroy the life of a patient who labours under renal disease, and it will be often more prudent to trust to the means which we possess of palliating his sufferings than run the risk of shortening his life in the endeavour to obtain a cure."

After a good description of the operation of lithotomy, we are carried by Sir Benjamin into the consideration of its dangers; and the most pressing of them is summed up in these words:

"All that I have been able to observe for many years past has confirmed me in the opinion, that an incision of the prostate extending into the loose cellular texture surrounding the neck of the bladder is replete with danger to the patient. Such a division of parts is never necessary where the calculus is of moderate dimensions, but it cannot be avoided where it is of a very large size; and hence the extraction of stones of this description can never be accomplished without a great probability of the patient not surviving the operation." (p. 327.)

This view of the case, as to the common cause of failure, is confirmed by the experience of Dupuytren. "To determine," says he, "why one in five or six die, it is necessary to ascertain what are the ordinary causes of death, and we find that among those who die after lithotomy, more than half, near three fifths, perish from inflammation about the bladder, the cellular tissue of the pelvis, the kidneys, &c." But what are the causes of those accidents? Sometimes we find the neck of the bladder and the left half of the prostate largely and neatly divided by the cutting instrument, the incision extending into the cellular tissue which surrounds the fundus and the sides of the bladder. "In fact these large incisions determine those inflammations, because they pass the limits of the neck of the bladder and that of the prostate, and because they allow the urine to come in contact with the pelvic cellular tissue."

The danger of large incisions is not a new discovery; we do not owe it either to Scarpa or Chaussier, though they may have more particularly pointed out the reason. It was a matter of long and very warm discussion between Lecat, Louis, and Frère Côme. The former composed three volumes to show the advantage of small incisions.

Now as to the extent of the incision there is much to be said in another point of view, the proportion of the bulk of the calculus to the incision. On the dead body of a child an incision of the prostate to the amount of three lines will allow of the extraction of a calculus of eight lines diameter. In a man of twenty an incision of six lines through the substance of the prostate will permit us to extract a calculus of fifteen lines diameter. In a man of forty an incision of eight lines will admit a cal-

* Prize Essay on the comparative advantages of Lithotomy and Lithotrity. (Ed. Med. Journ., No. 150.)

culus of eighteen to twenty lines to pass. Many apparently brilliant lithotomists are very unsuccessful, and we believe this is owing to the fact that they make an incision *so free* as to allow of the easy and immediate extraction of the stone; these free incisions pass beyond the limits of the prostate, penetrate beyond the pelvic fascia, and open a communication into the pelvic cellular tissue. All goes on well for forty-eight hours or more, and in as much more the patient dies, because the operator has followed the rule to cut the prostate gland "*through the whole extent of its lateral lobe*," and something more. It is surely quite time that the rule *not to cut to the extreme limit of the prostate* should be universally admitted and acted upon. It is true there are some cases where such an incision will not make the necessary space for the passage of the calculus, but they are not many; and as soon as the operator has satisfied himself of that by passing his finger into the bladder, he should enlarge the opening into the bladder by incising to a sufficient extent the opposite lobe of the prostate. Whether this be done in the ordinary way with the bistoury, or whether by the Celsian method,* but with other instruments, as practised by Dupuytren, is a matter upon which we cannot enter here; it is for the principle alone we contend, that one of the most fertile sources of evil in the operation of lithotomy is the extent given to the incision through the prostate.

We now proceed to that part of the volume which relates to the value of the operation of LITHOTRITY; and coming as it does from a person who has considerable experience on this much-debated subject, it is entitled to our most serious attention. It will assist us in estimating the confidence to which are entitled the opinions of those persons who would use lithotrity in every case, and of those who would reject it in all.

So much incorrect information exists on the subject of lithotrity, that we may be pardoned in offering a few remarks as to the history of the operation. First, it is a mistake to carry it back to the time of Celsus. The sentence so often quoted does not apply to lithotrity, but to lithotomy, when the stone is too large to be extracted by the wound: "Uncus injicitur calculo, ut facile eum conclusum teneat, ne is retro revolvatur; tum ferramentum adhibitur . . . quod admotum calculo . . . ictum fendit," &c. This practice might be carried back long before Celsus. Ammon of Alexandria being unable on many occasions to extract the stone through the wound, broke it down with a sculptor's chisel, and was therefore called Lithotomos. Whether Alsaharavius performed the operation in the twelfth century we cannot tell; certainly he described it: "Accipiatur instrumentum subtile, quod nominat *Mashaba rebilia* et suaviter intromittatur in virgam, et volve lapidem in medio vesicæ, et si fuerit *mollis* frangitur et exibit; si vero non exiverit cum iis quæ diximus oportet incidi," &c. C. F. Martius (Revue Med., July, 1837) extracts the following statement from an Arab author's disquisition on precious stones: "Un precieux avantage du diamant dont parle Aristote, et que l'expérience confirme, c'est son usage dans les affections calculeuses. Quand un malade est atteint d'une pierre soit dans la vessie, soit dans l'urètre, on prend un petit diamant, qu'on fixe à l'extremité d'une petite

* "Cum jam eô venit (calculus) ut super vesicæ cervicem sit, juxta anum incidi cutis plagâ lunatâ usque ad cervicem vesicæ debet, cornibus ad coxas spectantibus paululum."

tige metallique de cuivre ou d'argent; on l'introduit dans l'organe qui contient la pierre, et on la reduit par un frottement répété." Ebn-ab-Harrar states that he employed this plan on a servant in a case of very large calculus; he reduced it to so small a size that it could be expelled with the urine. Antonius Benivienius, who died in 1502, in a work intituled "Antonii Benivienii Florentini Medici ac Philosophi, de Abditis Nonnulis ac Mirandis Morborum et Sanationum causis liber," published after his death in 1506-7, mentions a case of a woman who for some days was affected with retention of urine, "propterea quod ipsius URINÆ ITER calculo obstrueretur." The disease not having yielded to any of the means tried, the author performed an operation, which is described in these terms: "Uncum calculo injicio, ne scilicet concussus iterum *in vesicam revolveretur.* Tum ferramento priore parte retuso calculum ipsum percutio, donec sæpius ictus in frusta comminuitur." The patient was cured as soon as the fragments came away with the urine.

Haller alleges that an instrument of three branches, and a central perforator, was used by Sanctorius. We think it cannot be denied that the monk of Citeaux relieved himself of a calculus by a percuting instrument. We then come to Martin, Gruithuisen, and Elderton, with whose merits all are familiar, to Leroy, Civiale, and others, whose claims to priority are hardly yet decided, though there is no doubt that with Civiale rests the priority of operating on the living subject.

All the known plans of operating resolve themselves into the following methods: successive perforations—breaking down by crushing—wearing away from the circumference to the centre. 1. To the first plan we may bring the methods of Sanctorius according to Haller and that of Gruithuisen. It is practised with the three-pronged instrument, and it is relative to the priority of invention of this instrument, that MM. Civiale and Leroy have so long been at issue: this was the method employed between 1824 and 1830. 2. *Crushing* either by pressure or percussion: a defective instrument for this purpose was invented by Amussat in 1822. An instrument little more convenient was the *brise-coque* of Heurteloup, presented in 1828. These were followed by two other instruments destined to change the character of lithotrity, the *brise-pierre articulé* of Jacobson, and the *percuteur* of Heurteloup; the first acts by pressure, the second by percussion, but it has been modified to act by pressure also. 3. The third plan, by *excentric destruction,* was invented to get rid of the difficulties and injuries of fragments, but it has not succeeded; it is that of Martin, Meyrieux, Tanihon, and Rigol.

We have now to enquire into the circumstances which justify us in having recourse to the operation: we will, in the first place, quote Sir B. Brodie's opinion; in the second, we will venture to give our own.

"It would be a great error to represent it as preferable on all occasions, but it is so in a great many instances; and I shall next endeavour, as a guide for your future practice, to explain by what signs you may distinguish from each other the cases to which it is applicable, and those to which it is not. In boys under the age of puberty, lithotomy is so simple, and so generally successful, that we ought to hesitate before we abandon it for any other kind of operation."

In this way more than half the cases are turned over to the old operation.

"There is also a manifest objection to lithotrity in these cases on account of the small size of the urethra, which is such that it would not admit of the introduction of instruments of sufficient strength to crush a calculus of more than moderate dimensions. .......... In cases in which the calculus has attained a very large size, it is often difficult to seize it with the lithotrity forceps; the operation of crushing requires to be repeated a great number of times, so that many weeks may elapse before the cure is accomplished; a larger quantity of fragments is left in the bladder, of which the necessary consequence is a great liability to inflammation of the mucous membrane, and of course the inconvenience produced by the passage of the fragments along the urethra is multiplied as compared with what happens when the calculus is smaller. These circumstances form a sufficient objection to the operation of lithotrity in these cases. It is true that they are unfavorable cases for lithotomy also, but I have little doubt that the latter method is the safer of the two. . . . . . . The operation of lithotrity is not well adapted to those cases of enlargement of the prostate gland in which the patient is *enabled* [qy. *unable?*] to empty the bladder by his own efforts, unless the calculus be of a small size, so that there may be no great difficulty in washing the minute fragments into which it has been crushed, out of the bladder through a large catheter. There is also another objection to the operation in some cases of enlargement of the prostate, namely, that the tumour which projects from it into the cavity of the bladder makes it difficult to elevate the handle of the forceps sufficiently to seize the stone easily in the usual manner." (pp. 375-6.)

"With the exception of such cases as those which have been enumerated, there are few to which this method of treatment may not be advantageously applied. It may be said that the exceptions are numerous, but they are the result chiefly of delay. If a patient seeks the assistance of a competent surgeon within six or even twelve months after a calculus has descended from the kidney into the bladder, the urine having remained acid, it will rarely happen that he may not obtain a cure by a single operation, and with so small an amount of danger that it need scarcely enter into his calculations. As time advances, the facility with which he can be relieved diminishes, and after the lapse of two or three years, especially if the urine has become alkaline, it is probable that the calculus will have attained such a size as to render the old operation preferable, and that the access of disease in the bladder or kidneys may render any operation hazardous. It would be absurd to say, and it would be unreasonable of human kind to expect, that an operation which has for its object to relieve them of a disease so terrible as that of a stone in the bladder, can be always free from inconvenience, and difficulty, and danger. Nevertheless, from what experience I have had, I am satisfied that the operation of lithotrity, if had recourse to only in proper cases, is not only much more successful than that of lithotomy, but that it is liable to fewer objections than almost any other of the principal operations of surgery." (p. 379.)

There is no difference of opinion on one point, and that is, that when a person is suffering from vesical calculus, if his general health and the state of the urinary organs be satisfactory, he should submit to some operation for its removal. It is now generally admitted that, spite of the brilliant prospects to which it gave birth, the operation of lithotrity is not applicable to every case; and as the fruitless attempts to relieve a patient from his suffering by this operation are not always free from danger, it is very desirable that the circumstances which justify a recourse to it may be placed on an intelligible basis. This Sir B. Brodie has sought to do; but unfortunately in the present state of our knowledge on the subject, it is very difficult to determine with any certainty the rules which should be applied, and which should never be based on any but a large number of observations fairly taken; and which if now laid down may require hereafter very considerable modification. Whether future observations shall restrain or extend the usefulness of this operation, it must still be considered as one of the most important discoveries of surgery in the present century.

In general terms the operation of lithotrity is indicated, in adult and aged persons of good general health, provided there be no great hypertrophy, or contraction, or paralysis, or increased sensibility of the bladder, or enlarged prostate, or diseased kidneys, provided also that there be a single and not very large or very dense calculus. It is objectionable in children, and where the texture and functions of the bladder are much changed, where there are many calculi, and where they are large or very hard. We do not mean to say that in many of the cases just enumerated lithotrity might not be practised with a chance of success. Numerous facts prove the contrary, but that is not the question ; what we want to know is whether in those conditions, lithotomy would not do better—and this might easily be proved. A large-sized stone is opposed to lithotrity; though we by no means deny that large stones have been successfully removed by the process, but a large stone generally supposes a small contracted bladder; when the bladder has not undergone those changes a stone of twenty or more lines in diameter has been removed. The shape of the stone is also important; the number of stones is no less important, because many stones presuppose many sittings; but if they are soft or friable this may not be the case. Civiale has crushed forty at a single sitting—some of them may have been fragments. Great density is an unfavorable circumstance; the softest are the triple phosphate, next come the lithic acid, lithate of ammonia; the hardest are the oxalate—by these, perforators have been blunted and percutors have been resisted.

The ordinary consequences of the operation of lithotrity must be materially influenced by the circumstances already detailed. In many cases a single sitting is enough. In the 14th observation of Civiale's Parallèle, twelve sittings were necessary for perforations alone. Many short sittings are less objectionable than a few long ones, the patient suffers less; but all this must depend upon the condition of the patient—some can bear without apparent inconvenience a sitting of a quarter of an hour, others are fatigued with two or three minutes. In some the detritus passes quickly, in others not till after twenty-four or forty-eight hours: in some not at all. When many fragments have passed the bladder must be examined with great care, because the smallest particle may become the nucleus of a new calculus. It is one of the objections made to the operation that it thus facilitates the reproduction of calculi. This is an inconvenience which cannot be denied; but whether it attaches more to lithotrity than to lithotomy—whether, in fact, when the bladder is supposed to be completely relieved, the recurrence of calculus is more frequent after lithotrity than lithotomy, is a question we cannot determine. M. Civiale says no.

The occasional consequences of the operation are fracture of instruments, perforation of the bladder, tearing off portions of the mucous membrane of the bladder, rupture of different parts of the urethra, peritonitis, infiltration from injury to the urethra, swelled testicle, articular inflammation, fever, nervous excitement, inflammation of the bladder, kidneys, or prostate. But of all the accidents attendant upon the operation, that which causes most embarrassment, if not danger, is the infarction of fragments of calculi in the urethra. Leroy admits that it happens in one case in four; and that he has either broken up or extracted at least six hundred fragments from the urethra (1837). This is certainly one of the greatest inconveniences with which the operation has to struggle. Sir B. Brodie thinks that the means of preventing this evil are very much in our own power. "A state of perfect repose in the recumbent posture, except when it is necessary to remove from one room to another, should be considered as indispensable after the operation; and I venture to say that when this rule is observed it will very seldom happen that the passage of the fragments along the urethra is productive of any serious inconvenience." Still it does happen, and in the hands of very prudent surgeons.

Let us say a few more words on the subject of this important operation. At its first introduction it was fondly thought that all the pain and danger of lithotomy could be avoided. Experience soon proved that the hope was delusive, by unfolding a long catalogue of accidents which, like those of lithotomy, often terminated in the death of the sufferer. To the early enthusiasm succeeded reflection and the necessity of a fair comparison of the new operation with that which it had been destined to supersede. But we are not yet in possession of the data necessary for this comparison. For the complete solution of the problem there should be two series of cases, placed as nearly as may be in the same condition as to age, general and local health, and physical condition of calculi: a similar number should be treated by each method and the results should be exactly followed. That has never been done, and probably never will be. A considerable experience, if not a rigorous statistical examination, has served to produce, in the minds of some of the best surgeons, the opinion that in the condition described as favorable for lithotrity, though it is not free from the chançe of accident, it is at all events much more favorable than lithotomy. We have also stated what may be expected from the operation in complicated cases; and we may remark that if lithotrity has so frequently failed it is to be attributed mainly to the unfavorable circumstances under which the operation is undertaken. On the one hand there is the natural tendency of inventors and enthusiasts to exaggerate the benefits to be derived from their invention: on the other hand there is the obstinate determination of many patients to reject a bloody operation, which they dread, to prolong this state of things. But when lithotrity has become an ordinary surgical operation, when more numerous observations, authentic in every particular, shall enable us to appreciate the limits of action of lithotrity, we have no doubt that its results will be much more favorable than they are at present: but to accomplish this its application must be restrained. We think there should always be a choice exercised; and as it is in the earlier periods of the disease that the most favorable conditions are presented, we may say that a comparison between these two methods of treatment can scarcely be instituted; they are applicable to different periods of the same disease. The cases which might be rejected by the lithotritist would fall into the hands of the lithotomist: that operation would then be more unsuccessful than it is at present, because it would only be used in complex cases.

